# Facility-led community based approach in Mamfe health district, Cameroon: a differentiated service delivery option in complex humanitarian settings

**DOI:** 10.1186/s12913-023-09323-9

**Published:** 2023-04-03

**Authors:** Enongene Julius Mekolle, Kate Ivo Keumami, Omeichu Agwenam Amadeus, Agbor Nyenty Agbornkwai, Ismaila Esa, Aseh Christian Chuyum

**Affiliations:** 1 Family Health International 360, Bafoussam, Cameroon; 2 Cameroon Baptist Convention Health Services, Bamenda, Cameroon

**Keywords:** Facility-led community-based approach, HIV, Differentiated service delivery, COVID-19, Conflict-affected setting

## Abstract

**Background:**

The government of Cameroon’s 2017 operational guidelines for the implementation of the “test and treat” strategy expressly incorporates and prescribes the differentiated service delivery (DSD) model with testing and treatment services being decentralized and task shifted at community level. However, express guidance on DSD approach in conflict situations, characterized by pressure on existing health systems remains a limitation. The outbreak of COVID-19 further confounded humanitarian responses for fear of spread. Facility-led community-based approach (FLCBA) was employed as a model of care in addressing DSD for HIV in conflict-affected settings within the COVID-19 context.

**Methods:**

A retrospective quantitative cross-sectional study was conducted in Mamfe District Hospital. Descriptive statistics was used to evaluate the implementation of FLCBA as a DSD model from April 2021 to June 2022 along the clinical cascades. Data were collected using a chart abstraction template from the respective registers. Analyses were done using Microsoft excel 2010.

**Results:**

In 15 months, a total number of 4707 (2142 males, 2565 females) people were screened for HIV and 3795 (1661 males, 2134 females) eligible individuals were tested. Out of the 11 targeted health areas, 208 (5.5%) new positive cases were identified, all (100%) of whom were linked to care and treatment. During this period, 61% (34/55) targeted missing clients were tracked through this means among which 31 were defaulters and 3 LTFU. Of the 196 target clients for FLCBA, eligible for viral load sample collection, 142 (72%) samples were collected.

**Conclusions:**

The FLCBA as an integral primary health care delivery package is an efficient and effective variant of DSD for conflict settings; however it requires bravery of health care providers.

## Background

The United Nations General Assembly adopted the Declaration of Commitment on HIV/AIDS in 2001, noting that “populations destabilized by armed conflict, humanitarian emergencies, and natural disasters, including refugees and internally displaced persons, and in particular women and children, are at increased risk of exposure to HIV infection” and that “national strategies that incorporate HIV/AIDS awareness, prevention, care, and treatment elements into pro-social programs” are necessary [1]. Reaffirming these pledges in the context of attaining universal access to HIV prevention, treatment, care, and assistance for vulnerable groups is part of the Political Declaration on HIV/AIDS from 2006 [2].

Differentiated service delivery (DSD), recommended for HIV treatment by the World Health Organization, since 2016 [3], previously referred to as differentiated care, is a client-centered approach that simplifies and adapts HIV services across the cascade to reflect the preferences, expectations and needs of people living with and vulnerable to HIV, while reducing unnecessary burdens on the health system [4]. Published evidence from the past decade show that DSD can: enhance client outcomes, including quality of care; ensure that the health system functions efficiently; enable the health system to refocus resources to those most in need [5]. The DSD was designed to improve retention in treatment within HIV programs in low-middle-income countries and has been globally adopted in national guidelines [5–8].

Cameroon, located in Sub-Saharan Africa has a population of about 27 million in 2021 [9], and an estimated 534,245 people living with HIV among whom 56.68% (302,822) were receiving ART by the end of June 2019 [10]. Though Cameroon currently ranks second with respect to HIV burden in Central and West Africa [11], the epidemiological burden of HIV in Cameroon is decreasing (from 5.5% in 2004 to 4.3% in 2011 to 2.7% in 2018) [12]. This progress is largely due to the government of Cameroon’s and its technical and financial partners’ concerted efforts in implementing evidence-based innovative strategies such as the rollout of PMTCT option B + since 2013, the “Test and Treat” strategy since 2016, increasing viral load uptake and coverage since 2007 [13]. The government of Cameroon’s 2017 operational guidelines for the implementation of the “test and treat” strategy expressly incorporates and prescribes the DSD model with testing and treatment services being decentralized and task shifted at community level [14].

Political unrest may have the greatest impact on the state’s ability to provide social services including health care. Historically political unrest has been documented to disrupt a country’s HIV/AIDS fight. However, some countries have successfully adapted DSD techniques to ensure non-rupture of care [15–17]. The current political crisis in Cameroon’s North West (NW) and South West (SW) regions is a particularly concerning recurrence of a long-standing issue which quickly evolved into a full-fledged armed conflict since 2016. The armed war has had the greatest impact on the SW area, with 15 of its 18 health districts seriously impacted [18]. Mamfe health district is one of the earliest and heavily affected localities by the conflict leading to forced migration. In addition to security concerns, continuous interruption of electricity resulting to poor use of communication devices by users, inability to travel freely due to erratic lock downs or expired identification documents compound difficulty of patient access to care and treatment. These constraints in access to care prompted the application or adaptation of DSD models for HIV in these complex humanitarian settings. In this paper, we review and evaluate the facility-led community-based approach (FLCBA) model executed between April 2021 to June 2022 to identify best practices in the implementation of integrated HIV DSD in fragile and conflict-affected settings and make recommendations for optimizing the approach in fragile and conflict-affected settings.

## Methods

### Study design

A retrospective quantitative cross-sectional study was conducted in Mamfe District Hospital, with 1,968 PLHIV on treatment, one of the 25 high volume health facilities in the South West region of Cameroon supported by Cameroon Baptist Convention Health Services. Descriptive statistics was used to evaluate the implementation of facility-led community-based approaches (FLCBA) as a DSD model from April 2021 to June 2022 along the clinical cascade. This included case identification, linkage to care of newly diagnosed clients, continuity of treatment, tracking of missed appointments, ARV defaulters and loss-to-follow-up (LTFU) and viral load sample collection led by the facility, to the community and feedback report to the facility. The FLCBA is akin to the mobile clinics strategy successfully executed and reported by Omam et al. in their work in the North West and South West regions of Cameroon [17]. Mamfe is a rural setting with some villages enclaved, thus DSD models focusing on clients’ needs were prioritized.

### Inclusion and exclusion criteria

We included all adults and children in the area of coverage.

### Study area

Two sets of teams were constituted according to Health areas and assigned targets. Team one (T1) included Mamfe, Kendem, Bachuo Akagbe, Kajifu and Tali health areas while Team two (T2) covered Eyumojock, Afap, Ekok, Kembong and Ogurang health areas.

### Service providers

Service providers of both teams were trained and had basic HIV clinical background. Each team was made up of 4 health personnel; a nurse (team lead), 1 community mobilizer, 1 counselor, 1 data clerk and a medical doctor providing oversight coordination of both teams and intermittent field interventions. The community mobilizer was involved in engaging and preparing the community stakeholders for the activity package. The counsellor and nurse screened the population and tested for HIV. The entire team was also responsible for tracking PLHIV who had missed their appointments, or who were LTFU and ensure their return to care. The nurse was responsible for ARV prescription and viral load sample collection. The team data clerk ensured all activities were documented accordingly in the respective log registers provided. Both team activities were boosted by an accessory team of psychosocial agents also responsible for contact tracing, tracking missed appointments, LTFU and viral load sample collection.

### Strategic planning and frequency of services

Prior to the week’s activity, the entire team met and mapped out communities to target for case identification using the existing catalogue of communities. 3–4 outreaches were planned each week to target 5–6 communities. Prior to community visit, the communities were notified and mobilized. In other cases, the team co-planned with partner organizations such as AMEF who are implementing partner for WFP covering nutritional needs of the communities. The partnership was beneficial because it drew a larger gathering enabling the provision of a comprehensive health package.

On the day of activity, teams set up in hubs within the visited community, with each member carrying out specialized roles of counselling, testing and delivery of results. All testing were carried out according to the national algorithm for HIV testing. Confirmed positive clients were linked to care, affiliating them to the closest primary health care facility. Another strategy employed by the team was use of existing community-based health centers. Test strips were made available to the local health facility chief of center, who notified the team in the event of a new case identified. The counselor and nurse followed-up to confirm, counsel, and link the client to care.

On a weekly basis, data on missed appointments, LTFU, clients pending or missed viral load appointments were pulled from the database locally. These data were disaggregated according to health area coverage by the teams, and according to psychosocial agent follow-up cohorts. These printout data were used to develop individual/ team weekly work plans for community tracking. Communities were revisited as often as the need requires for anti-retroviral therapy (ART) dispensation and viral load sample collection. However, for case identification, communities were re-visited once in 3 months. Blood samples were drawn according to the national guidelines. Samples were transported to the health facility in cool boxes; prepared, stored and safely evacuated for analysis in the reference laboratory. Samples were evacuated weekly.

Poor roads, bad communication network and erratic insecurity incidences from gunshots, kidnapping or forceful retention were the main barriers to reaching intended weekly target clients, locations and populations. Communities that could not be accessed on a planned date due to security concerns were rescheduled to be visited on a later date. With the support of community facilitators and community leaders who mobilized the communities, in addition to unique identification jackets, the teams were able to access most targeted communities.

### Data analysis

All daily data from the teams were captured on log registers and entered into Data manager software (DAMA) for synthesis and analysis using Excel version 9. The teams were responsible for confidentiality of all client information.

## Results

### Service delivery

#### Case identification and linkage to care of newly diagnosed clients during mobile clinics

Over 5 quarters, between April 2021 to June 2022, a total number of 4707 (2142 males, 2565 females) people were screened for HIV across all covered health areas. 3795 (1661 males, 2134 females) eligible individuals were tested. Team 2 tested the highest number of persons, 2153 (56.7%). Out of the 11 targeted health areas, 208 (5.5%) new positive cases were identified (Table 1). Team 1 recorded the highest number of positive cases 138/208 (66.3%). All newly diagnosed clients (100%) were linked to care and treatment.

**Table 1:** Case identification

Period	Teams	Number screened			Number tested for HIV			Number tested positive (new)		
		M	F	Total	M	F	Total	M	F	Total
FY^a^21, Q^a^3 (Apr-Jun 21)	TEAM 1	100	139	239	33	51	84	18	25	59
	TEAM 2	89	94	184	69	72	142	6	10	
FY21, Q4	TEAM 1	80	90	170	53	41	94	24	21	67
	TEAM 2	90	73	163	40	46	86	7	15	
FY22, Q1 (Oct-Dec 21)	TEAM 1	320	400	720	258	326	584	12	18	43
	TEAM 2	400	450	850	323	428	751	5	8	
FY22, Q2	TEAM 1	230	300	530	178	266	444	5	7	21
	TEAM 2	300	345	645	269	319	588	3	6	
FY22, Q3 (Apr-Jun 22)	TEAM 1	221	300	521	171	265	436	1	7	18
	TEAM 2	312	374	686	267	320	587	4	6	
	TOTAL	2142	2565	4707	1661	2134	3795	85	123	208

^a^*FY *fiscal year, *Q *quarter

#### Tracking of clients who missed appointment and LTFU

Out-of-facility individual care was used. Clients were tracked through phone calls and home visit using localization plans or searching by word-of-mouth, especially for clients who have migrated out of their usual locations. During this period, 61% (34 of 55) targeted missing clients were tracked through this means among which 31 were defaulters and 3 LTFU (Table 2).

**Table 2:** Tracking of missing clients

**Period**	**Tr-Curr**^a^** at Q**^a^** start**	**Teams**	**Target defaulters (D)/LTFU by quarter end**			**Defaulters tracked by quarter end**			**LTFU tracked by quarter end**			**Total**
			D	LTFU	Total	M	F	Total	M	F	Total	
FY^a^21, Q3(Apr-Jun 21)	1^st^ Apr-30^th^ Jun	TEAM 1	5	1	6	2	4	6	1	0	1	7
		TEAM 2	3	0	3	0	0	0	0	0	0	
FY21, Q4	1^st^ Jul-30^th^ Sep	TEAM 1	4	1	5	2	1	3	0	0	0	3
		TEAM 2	2	0	2	0	0	0	0	0	0	
FY22, Q1 (Oct-Dec 21)	1^st^ Oct-31^st^ Dec	TEAM 1	9	2	11	3	6	9	1	1	2	11
		TEAM 2	3	0	3	0	0	0	0	0	0	
FY22, Q2	1^st^ Jan-28^th^ Feb	TEAM 1	3	1	4	0	0	0	0	0	0	6
		TEAM 2	7	0	7	1	5	6	0	0	0	
FY22, Q3(Apr-Jun 22)	1^st^ April-30^th^ Jun	TEAM 1	9	1	10	4	3	7	0	0	0	7
		TEAM 2	4	0	4	0	0	0	0	0	0	
**TOTAL**			49	6	55	12	19	31	2	1	3	34

^a^*Tr-Curr *treatment current, *Q *quarter, *FY *fiscal year

#### Viral load sample collection at community level

Of the 196 target clients for facility-led community-based approach, eligible for viral load sample collection, 142 (72%) samples were collected (Table 3).

**Table 3:** Viral load sample collection

Period	Teams	Total eligible			Total sample collected		
		Target	M	F	M	F	Total
FY21, Q3(Apr-Jun 21)	TEAM 1	64	23	41	13	40	73
	TEAM 2	27	7	20	5	15	
	Total	91	30	61	18	55	
FY21, Q4	TEAM 1	40	25	15	15	14	36
	TEAM 2	17	12	5	2	5	
	Total	57	37	20	17	19	
FY22, Q1	TEAM 1	10	7	3	3	3	11
(Oct-Dec 21)	TEAM 2	7	2	5	2	3	
	Total	17	9	8	5	6	
FY22, Q2	TEAM 1	14	4	10	4	5	22
	TEAM 2	16	8	8	5	8	
	Total	30	12	18	9	13	
FY22, Q3	TEAM 1						^a^Missing data
(Apr-Jun 22)	TEAM 2						
TOTAL		195	88	107	49	93	142

^a^Not available at time of analysis

### Adaptations of DSD services to face the compounding COVID-19 pandemic

Our activities also responded to the need for awareness among people living with HIV to ensure they are protected from COVID-19. Prevention messaging was integrated into community-based activities during consultations. Protective materials including masks were shared with field staff where face-to-face meetings were required, as well as strict measures for all clients and health care workers to wear a face mask when providing or receiving services in the community and facility. Staff rotation roaster (shift system) was created to reduce crowding of health workers on duty so as to reduce the risk of transmission.

We adapted delivery of HIV testing, and initiation; Community based testing, verification and initiation was reinforced since client’s fear reaching the facility. We also expanded the use of HIV self-testing. We conducted rapid assessments and counseling support services virtually through voice calls and messaging.

In a bid to increase out-of-facility delivery of services to ensure that life-saving drugs continued to reach people with HIV, during the humanitarian crisis for NW/SW complicated by COVID-19, our programming of HIV activities were adjusted to support home delivery of antiretroviral therapies, testing services and viral load sample collection. Measures included reinforcement use of community ART groups, community-based organizations, encouraged family model of ART dispensation, free COVID testing for suspected cases and their contacts, extend ART prescriptions and refills, providing 3–6 months multi-month dispensation where stock permits and spacing out appointment visits.

## Discussion

Instances of conflict or natural disasters frequently result in the destruction of health and community structures, other services, and infrastructure, making it challenging to provide HIV services to internally displaced people and those affected by humanitarian crises [19]. Our encounter was as described. However, we won’t achieve zero new HIV infections, zero discrimination, and zero AIDS-related deaths unless HIV is addressed when assisting those affected by humanitarian situations [19]. Different DSD models are being applied to broaden ARV access in challenging areas [7]. These include, but are not limited to, community ART groups, mobile clinics, patient-led community ART distribution sites, and home-based ART dispensation [20–23].

Despite being reportedly expensive and logistically challenging, community-based models in the form of mobile clinics have been used successfully in conflict-affected settings to provide primary healthcare [24] and HIV services in remote, hard-to-reach settings [25, 26], including the Cameroonian setting [17]. A competent workforce was used in our FLCBA paradigm of care for DSD (Table 2). This model has been demonstrated to be an efficient way to reduce program retention hurdles and produce good rates of virologic suppression; as a result, it is suitable for DSD HIV services in bringing care closer to communities on a regular basis [27].

The teams used FLCBA to test positive, a total of 5.5% of people in the district’s 11 targeted health areas. This number is higher than the national prevalence (2.7%) and regional prevalence (3.2% SW), albeit the South-West prevalence primarily accounts for the situation in urban areas. This demonstrates how crucial it is to have evidence-based DSD for HIV particular to these insecure, isolated environments. All clients who received a new diagnosis were connected to care and therapy.

The high levels of insecurity in these places, the distance that PLHIV must go to receive ARV, and the cost of transportation have all been proven to contribute to gaps in retention to care and unsuccessful links to care [17]. To circumvent this obstacle, our teams made community-bound trips and successfully located 61% of missing clients using FLCBA. Though due to exceptional unsafe circumstances, some journeys had to be cancelled. Our strategy had a flaw in that we couldn’t properly trace every defaulter and LTFU back to ART. Due to a variety of factors, including inaccessible phone contacts throughout their tracking, our teams were unable to fill in these gaps. Poor network deep in the rural communities, a lack of electricity to power phones, or a lack of phones were some of the other related causes. Additionally, they found that PLWHA frequently unexpectedly migrated from their former residence in response to unpredictable insecure incidents or in pursuit of a better way of life, despite the lack of a precise quote on the next location. To overcome the issue of distance to care, partnerships with satellite health clinics in rural areas through the provision of anti-retroviral medications acted as hubs to serve some clients in the extremely remote settlements in the bushes.

Between clinical sites (urban, peri-urban, and rural) and centralized laboratories, viral load monitoring calls for coordinated transportation of specimens and results [28–30]. With the help of our facility-led community-based strategy, we successfully identified and collected viral load samples for 72% of the targeted eligible clients in order to facilitate the current constraints. Reasons for the gaps mirrored those of linkage. However, in carrying this out, we ran into a number of obstacles: clients missed community appointments since some prioritized farming schedule. Additionally, it could take longer to return samples to the facility for preparation and storage before transportation to the central laboratory, making cold chain handling of samples in transit troublesome. Also, blood samples were drawn in below-average quantity. Plans for the transportation of samples for analysis have been thwarted by lockdowns. Additionally, the destruction of the medical institution resulted in the loss of samples and significant difficulties with sample processing and storage.

Using the words of the UNHCR High Commissioner, he declared that “Programs aiming to reduce the stigma and discrimination faced by refugees, IDPs, and migrants need to be implemented at all levels, at the national level, they need to be included in HIV/AIDS National Strategic Plans, policies, and funding proposals.” The results thus far are depressing, he said. Since 2006, fewer refugees have been included in HIV National Strategic Plans. In 2008, only 32 of the 46 nations hosting more than 5,000 refugees had National Strategic Plans that could be examined. 14 of them, or roughly 44%, made no mention of refugees [31]. Our country health systems will gain immensely from unique national HIV policies and strategies geared to humanitarian circumstances.

In this paper, we attempted to capture the experiences, results and challenges with implementing facility-led community-based approach as a model of differentiated service delivery for PLWHA in humanitarian settings. The targets with respect to various aspects of the HIV cascades are specific to the FLCB approach and so do not reflect the global figure. A more comprehensive evaluation may be required to assess with completeness, the potentials of all approaches employed to provide patient-centered care to this underserved population.

## Conclusions

The facility-led community-based approach was used to deliver decentralized HIV care for IDPs in conflict-affected communities at the height of ongoing socio-political unrest in Cameroon, which was made worse by the COVID-19 pandemic. However, there is a dearth of evidence regarding the model of care for HIV DSD in conflict settings’ efficiency and effectiveness. FLCBA is viable since it is an integrated package of services streaming from the hub medical facility, even though it can call for bravery on the part of healthcare professionals in the face of the insecurity that characterizes a typical conflict setting. We recommend a comprehensive comparative approach across all existing DSD models or randomized controlled trials to be conducted on the use of FLCBA in conflict settings as a model of care in HIV DSD. Despite the potential expense of moving medical personnel to remote locations, the strategy appears to be cost-effective when compared to financial and security cost on patients.

## Data Availability

The datasets used and/or analyzed during the current study are available from the corresponding author on reasonable request.
